# Supplementary material for: Synthetic Immunological Niche Reveals Early Immune Dysregulation and Stratifies Therapeutic Response in Type 1 Diabetes
Source: bioRxiv. 2026 Mar 10:2026.03.06.710219. Preprint. [Version 1] doi: 10.64898/2026.03.06.710219 (PMC13060903; doi:10.64898/2026.03.06.710219)
Supplement: Supplement 1 [file media-1.pdf]

Supplementary Materials

Synthetic Immunological Niche Reveals Early Immune Dysregulation and Stratifies Therapeutic Response in Type 1 Diabetes

Jyotirmoy Roy[1], Yifei Jiang[1], Runbo Mao[2], Jessica L. King[1], Amod Talekar[1], Lillian Holman[1], Haoxuan Zeng[2], Xin Luo[2], Peter Sajjakulnukit[3], Brianna Ha[4], Kai Liu[2], Elizabeth J. Bealer[1], Laila M. Rad[1], Shahzad Sohail[1], Antonio Holmes[1,5], Bryan Wonski[1], Kathryn Kang[1], Dominik Awad[3,7], Aaron H. Morris[1], Costas A. Lyssiotis[3,6,7], Jie Liu[2], Lonnie D. Shea[1,4,8,*]

Affiliations:

[1] Department of Biomedical Engineering, University of Michigan, Ann Arbor, USA

[2] Gilbert S. Omenn Department of Computational Medicine & Bioinformatics, University of Michigan, Ann Arbor, USA

[3] Department of Molecular & Integrative Physiology, University of Michigan, Ann Arbor, USA

[4] Department of Chemical Engineering, University of Michigan, Ann Arbor, USA

[5] Michigan Institute for Clinical & Health Research, University of Michigan, Ann Arbor, USA

[6] Department of Internal Medicine, Division of Gastroenterology and Hepatology, University of Michigan, Ann Arbor, USA

[7] Rogel Cancer Center, University of Michigan, Ann Arbor, USA

[8] Department of Surgery, University of Michigan, Ann Arbor, USA

* Corresponding author: ldshea@umich.edu

a Myeloid Gating

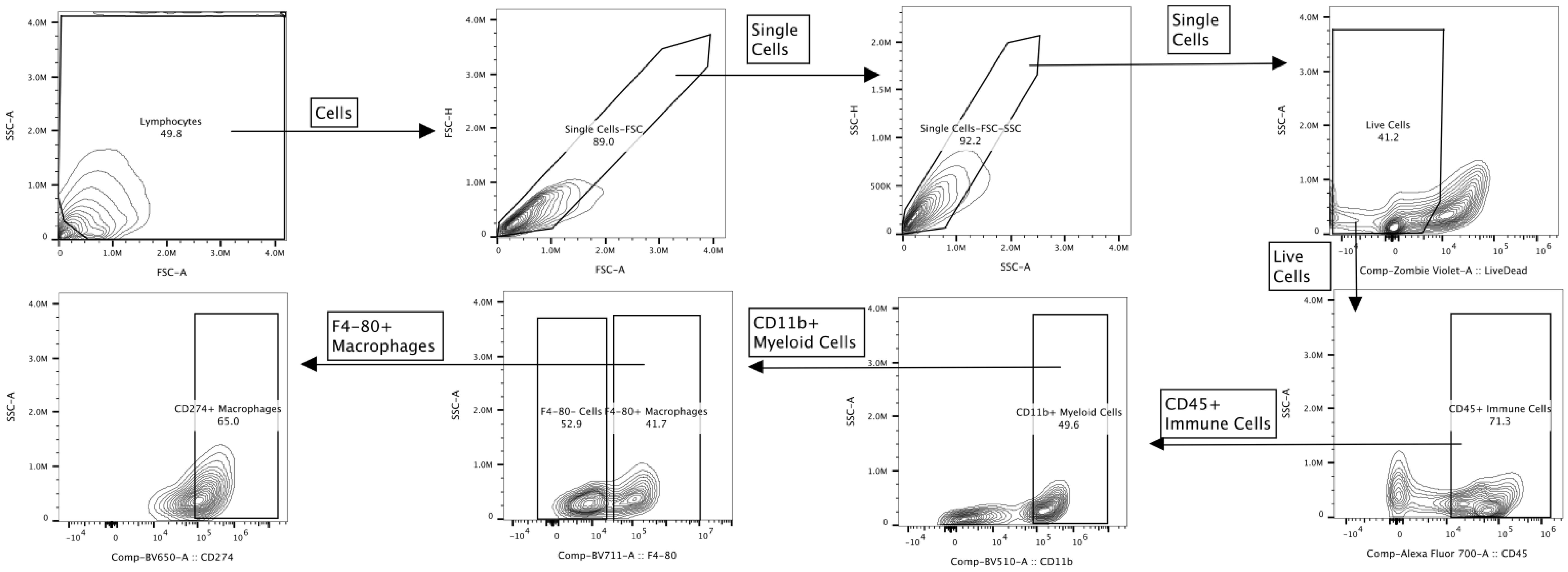

b Lymphoid Gating

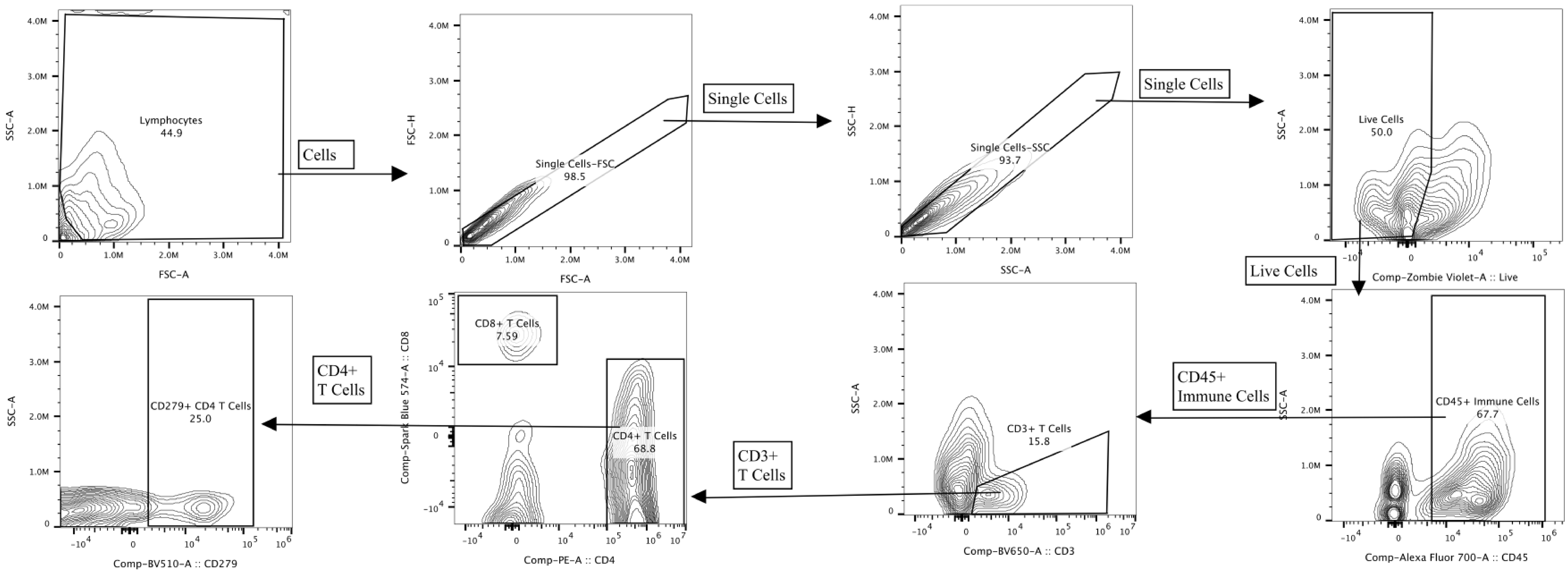

Figure S1: **Flow Cytometry Gating Strategy-** Flow cytometry gating strategy for **a)** Myeloid and **b)** Lymphoid panel

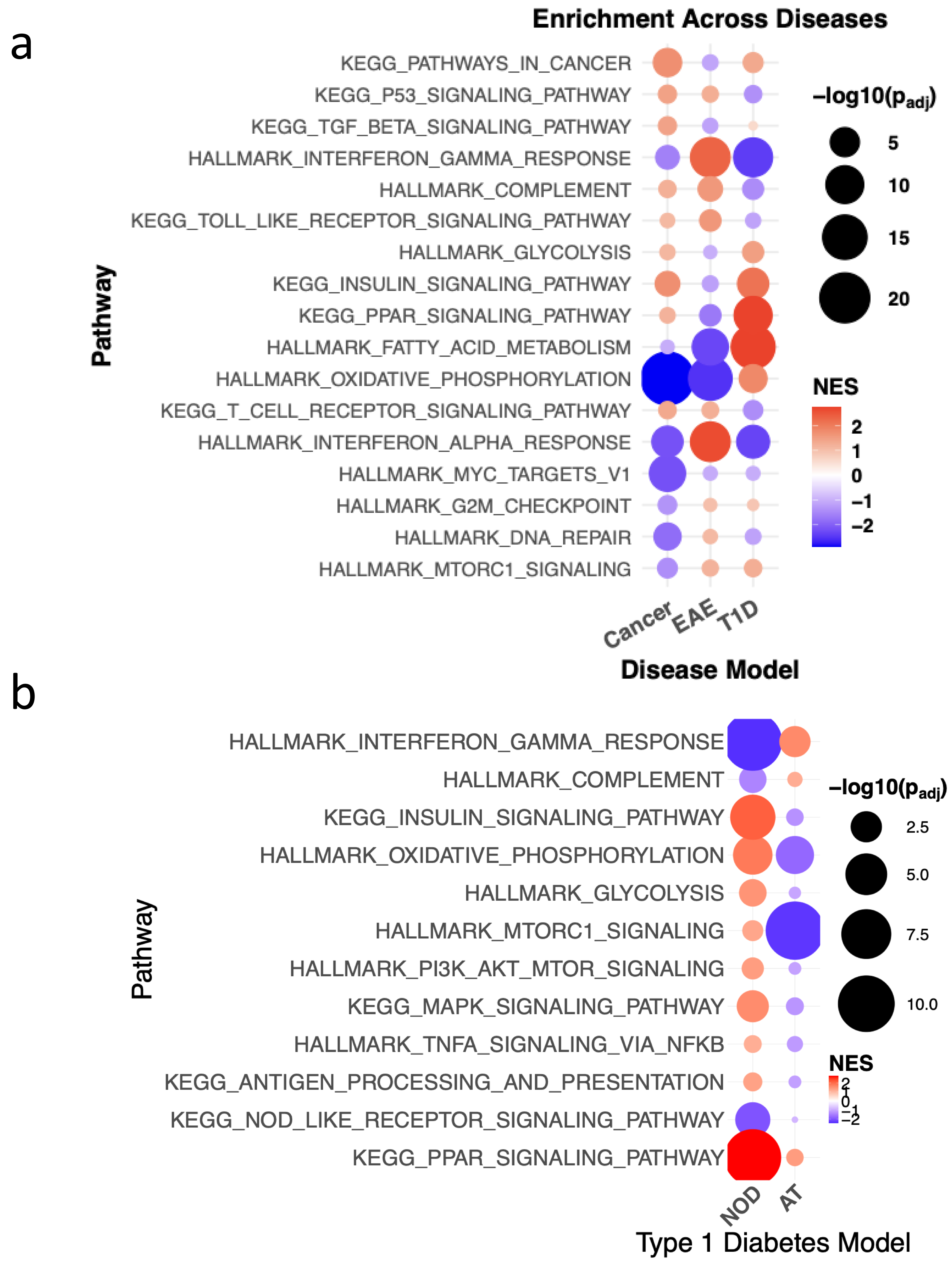

Figure S2: **Geneset Enrichment Analysis of IN transcriptomics-** Dotplot showing Normalized Enrichment Score and significance for selected pathways for- **a)** Early Stage NOD T1D vs 4T1 Breast Cancer and Experimental Autoimmune Encephalomyelitis. inflammation disease comparison **b)** Early Stage NOD vs Adoptive Transfer (AT) T1D models

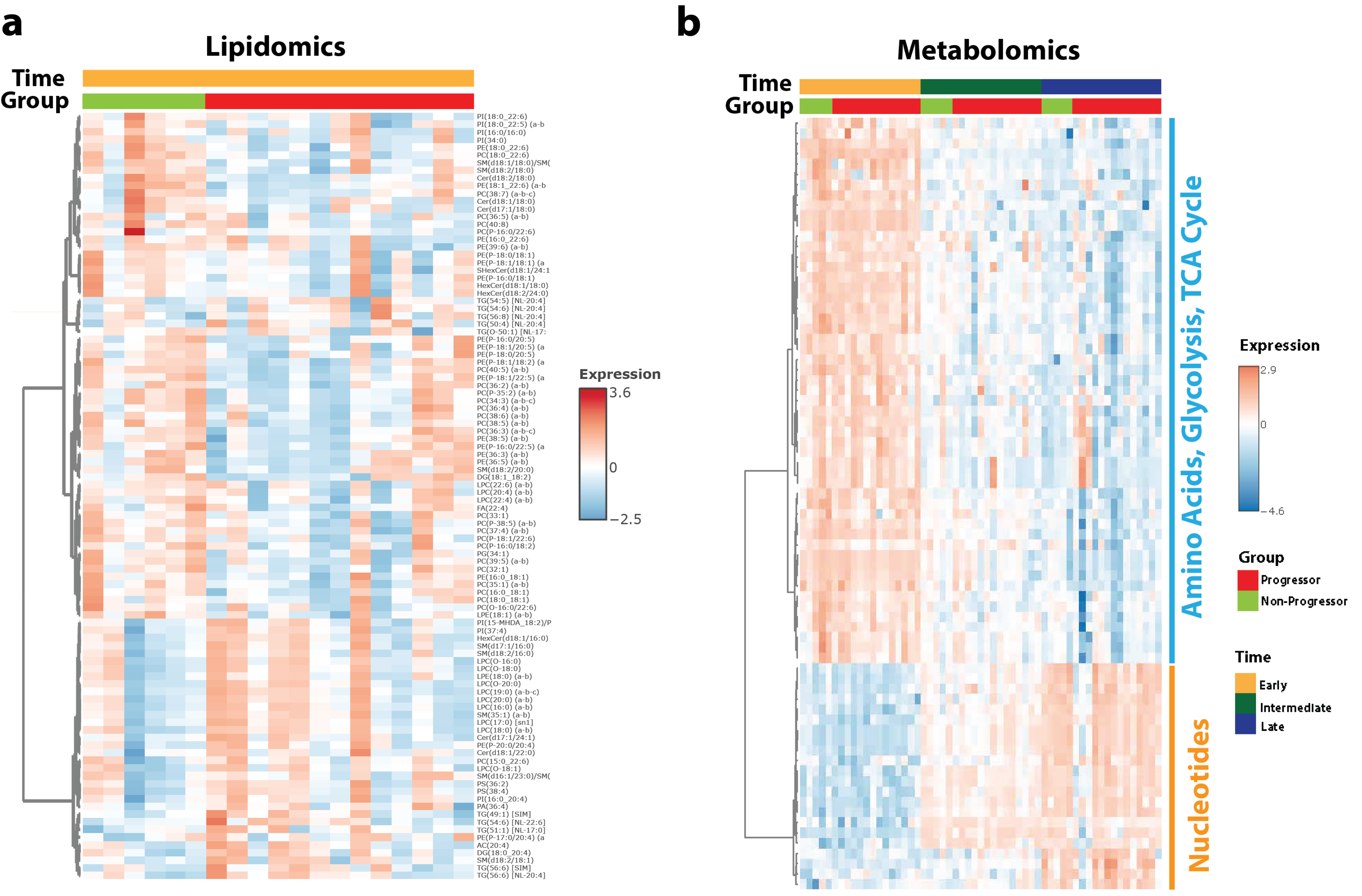

Figure S3: **Lipidomic and Metabolomic profiling of the Immunological Niche- a)** Lipid profiles at the early stage between progressor(n=13) vs non-progressors (n=6) **b)** Polar metabolite profiles at the different T1D stages between progressor(n=14) vs non-progressors (n=4)

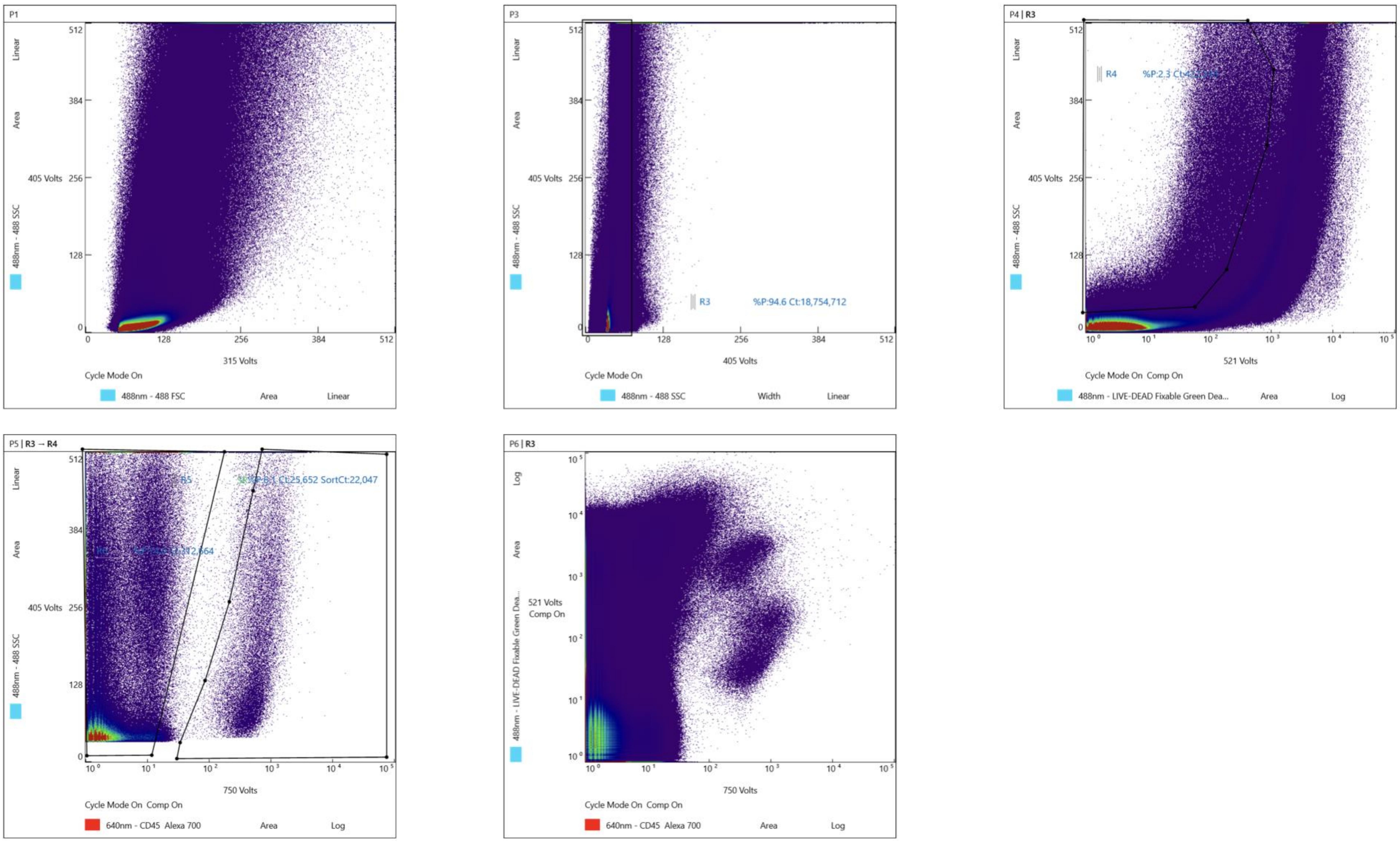

Figure S4: **Gating Strategy For NOD Pancreas Immune Cell Sorting using FACS-** Immune cells were sorted based of Live CD45+ gating

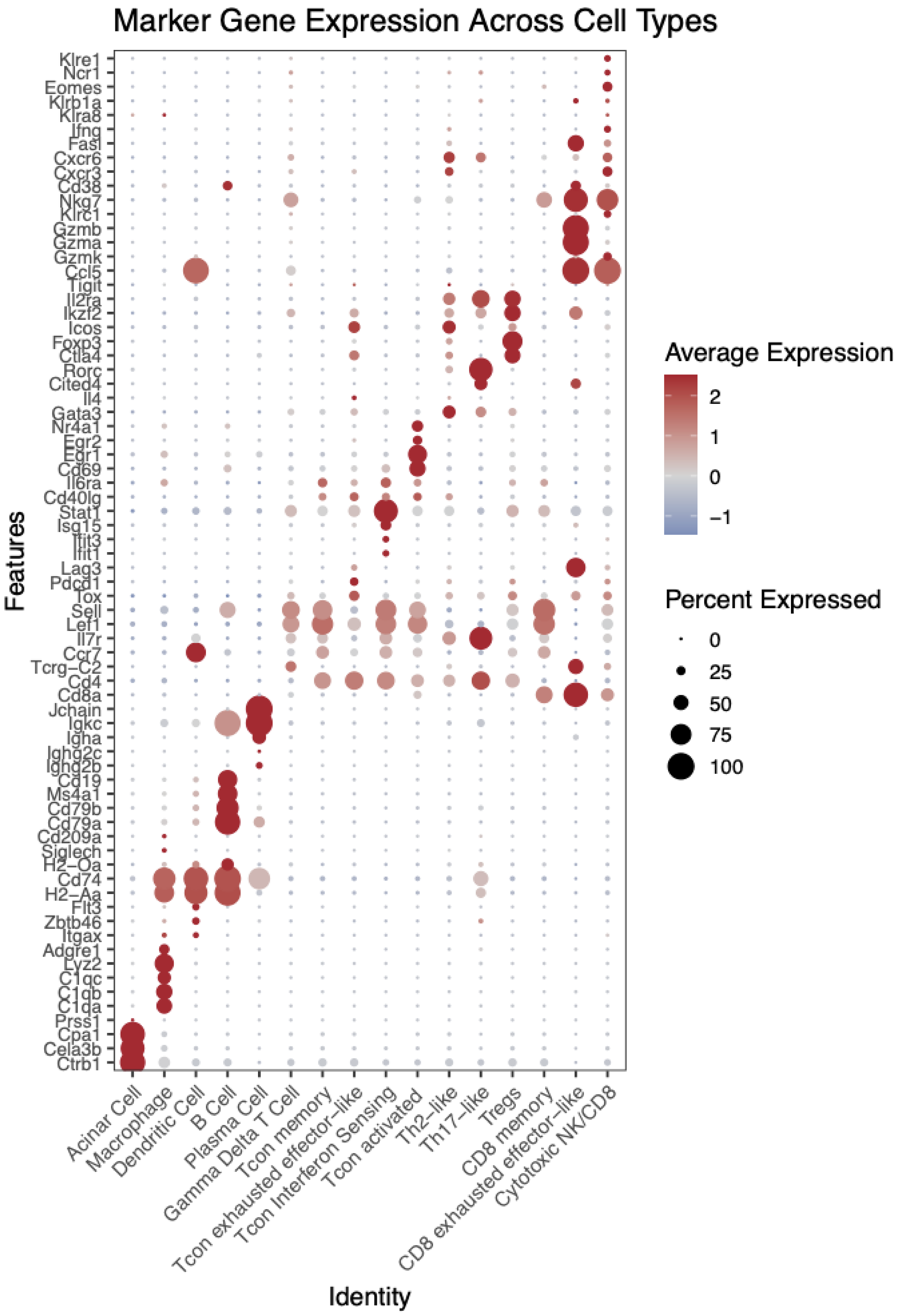

Fig. S5. **Dot plot of canonical marker genes used for annotation of pancreas cell types-** Scaled expression levels and frequency of canonical genes are shown for each identified NOD pancreatic cells

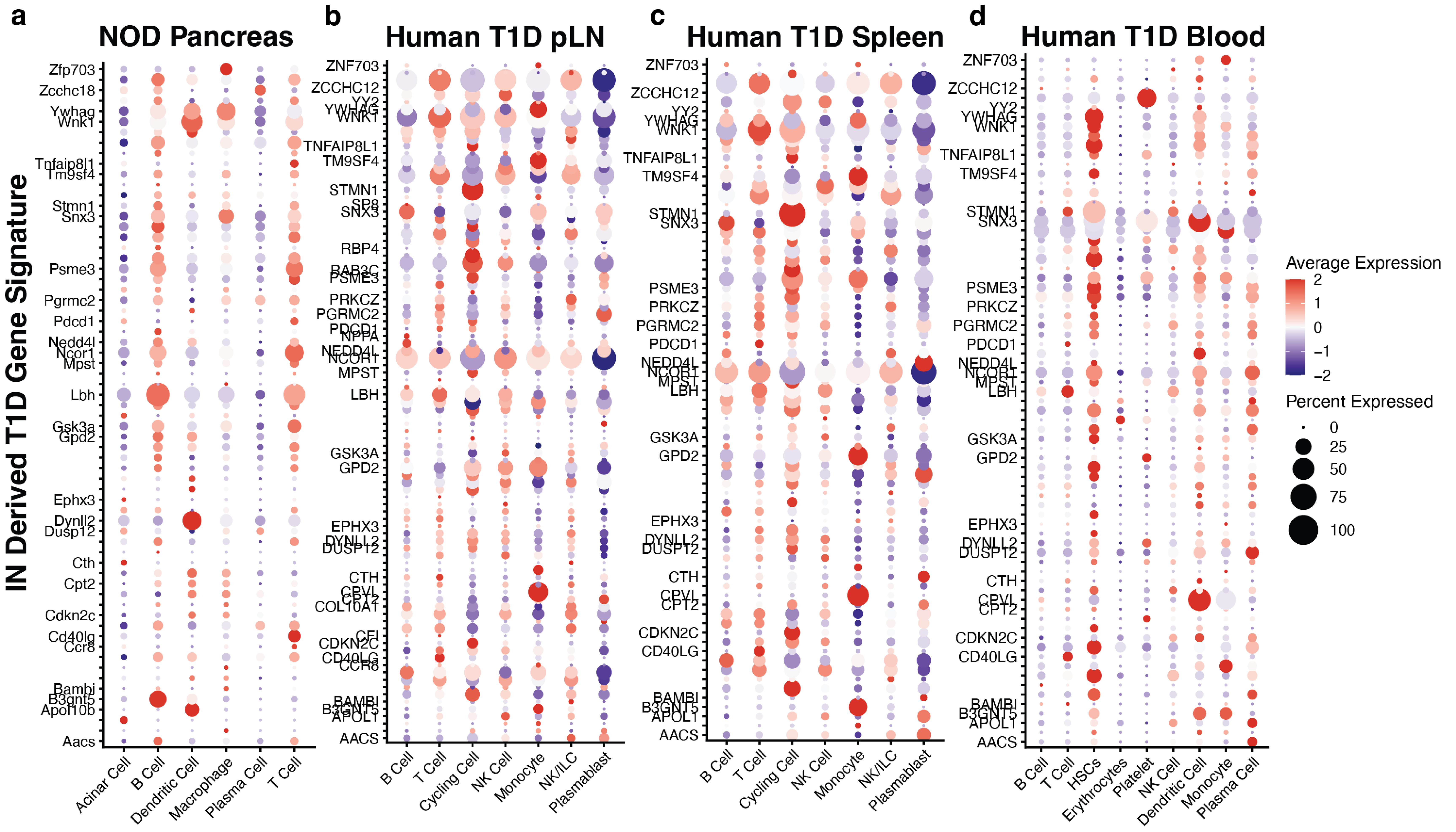

Fig. S6. **Mapping of IN derived T1D gene signature.** Scaled expression levels and frequency of T1D gene signature (or their human orthologs) for **a)** NOD Pancreas, and **b)** Human pLN, **c)** spleen, **d)** PBMCs from T1D and non-diabetic individuals

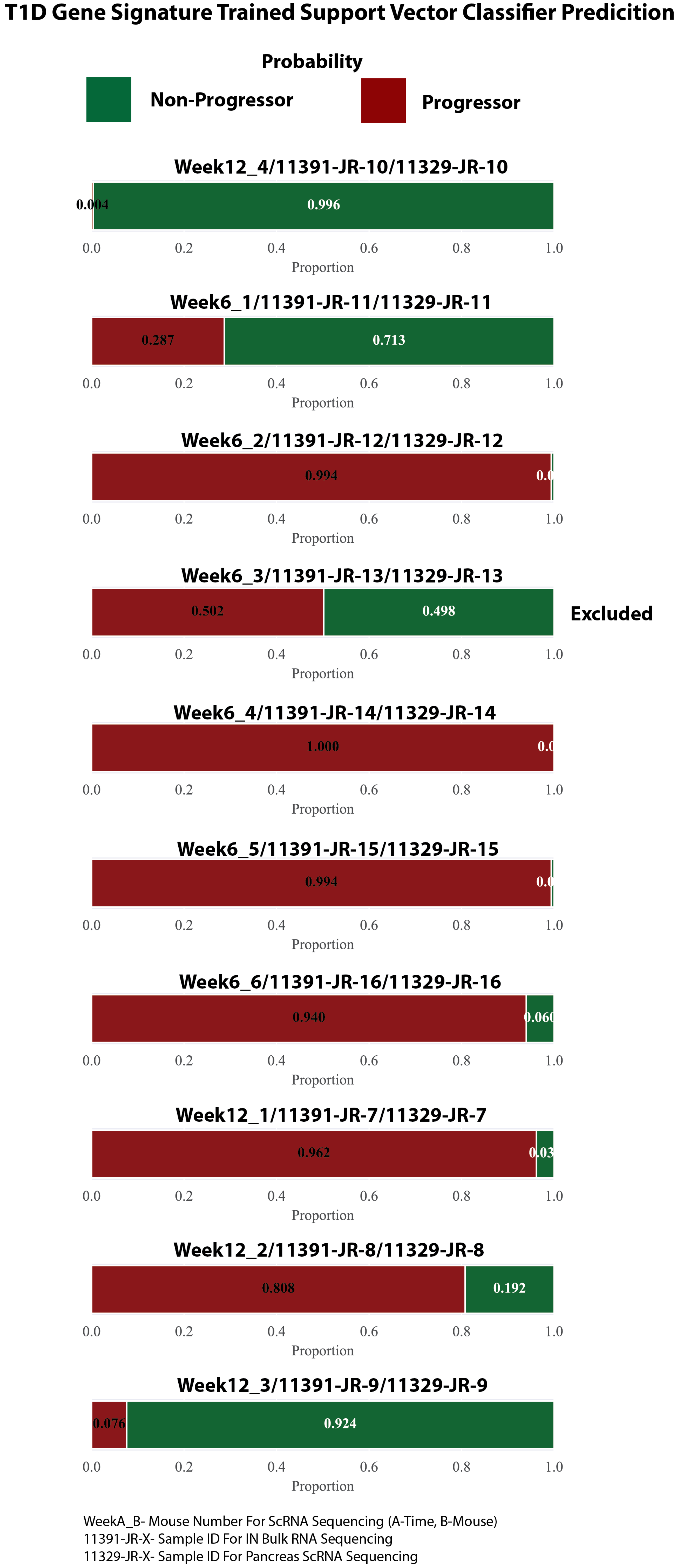

Fig. S7. Prediction of NOD as Progressor vs Non-Progressor Using IN Derived T1D 100 Gene Signature. Probability of being Progressor as predicted by the Support Vector Classifier trained on IN Derived T1D 100 Gene Signature. Samples classified with >60% probability were taken for downstream analysis of the pancreas single cell RNA sequencing data
